# Transcranial Alternating Current Stimulation: A Potential Risk for Genetic Generalized Epilepsy Patients (Study Case)

**DOI:** 10.3389/fneur.2016.00213

**Published:** 2016-11-28

**Authors:** Daniel San-Juan, Carlos Ignacio Sarmiento, Axel Hernandez-Ruiz, Ernesto Elizondo-Zepeda, Gabriel Santos-Vázquez, Gerardo Reyes-Acevedo, Héctor Zúñiga-Gazcón, Carol Marina Zamora-Jarquín

**Affiliations:** ^1^Department of Clinical Research, National Institute of Neurology and Neurosurgery, Mexico City, Mexico; ^2^Department of Basic Sciences and Engineering, Autonomous Metropolitan University Campus Iztapalapa, Mexico City, Mexico; ^3^Superior School of Medicine, National Polytechnic Institute, Mexico City, Mexico; ^4^Medicine Academic Unit, Autonomous University of Nayarit, Tepic, Mexico; ^5^Department of Medicine and Nutrition, University of Guanajuato, León, Mexico; ^6^Department of Clinical Sciences, University of Monterrey, San Pedro Garza-García, Mexico; ^7^Institute of Neuropsychology and Neuropsychopedagogy, Mexico City, Mexico

**Keywords:** transcranial alternating current stimulation, neuromodulation, juvenile myoclonic epilepsy

## Abstract

Transcranial alternating current stimulation (tACS) is a re-emergent neuromodulation technique that consists in the external application of oscillating electrical currents that induces changes in cortical excitability. We present the case of a 16-year-old female with pharmaco-resistant juvenile myoclonic epilepsy to 3 antiepileptic’s drugs characterized by 4 myoclonic and 20 absence seizures monthly. She received tACS at 1 mA at 3 Hz pulse train during 60 min over Fp1–Fp2 (10–20 EEG international system position) during 4 consecutive days using an Endeavor™ IOM Systems device^®^ (Natus Medical Incorporated, Middleton, WI, USA). At the 1-month follow-up, she reported a 75% increase in seizures frequency (only myoclonic and tonic–clonic events) and developed a 24-h myoclonic status epilepticus that resolved with oral clonazepam and intravenous valproate. At the 2-month follow-up, the patient reported a 15-day seizure-free period.

## Introduction

Transcranial alternating current stimulation (tACS) is a neuromodulation technique that consists in the external and non-invasive application of oscillating electrical current wave. It uses any wave form possible, such as sinusoidal or rectangular current shape waves (1). This stimulation technique aims to interfere with ongoing oscillations in the brain and induces changes in cortical excitability. tACS modulates cerebral rhythms applied at specific electroencephalographic frequencies (0.1–80 Hz) and in the “ripple” range (140 Hz) (1–4). Therefore, if tACS is effective for cortical neuromodulation, its effect appears to be generalized to all the brain, instead of localized to a single cortical area (5). It has been postulated that tACS cortical effects are dependent on the stimulus intensity and frequency (2, 6); frequencies of 1–5 Hz are excitatory similar to anodal transcranial direct current stimulation (tDCS) when 1 mA intensity is used, while those higher than 10 Hz and low intensity of 0.4 mA are inhibitory such as cathodal tDCS (6–8).

Juvenile myoclonic epilepsy is a generalized epileptic syndrome, which is conceptualized as originating at some point within the cerebral cortex, and rapidly engaging, bilateral distributing networks (9). Even though they are considered to be benign, it is estimated that up to 10–20% are refractory to pharmacological treatment (10), justifying the search for alternative treatments (11).

Neuling et al. (12) report that tACS can regulate brain oscillations in a frequency-dependent manner, thus, a clinical use can be given to this neuromodulation technique. Nonetheless, evidence regarding safety parameters and long-lasting after effects with tACS are still lacking. The objective of this paper is to present a clinical case regarding a patient with pharmaco-resistant juvenile myoclonic epilepsy who received treatment with tACS and developed serious adverse effects. This clinical trial received approval from the Bioethics and Research Committees of the National Institute of Neurology and Neurosurgery in Mexico City.

### Patient Case

We present the case of a 16-year-old female student, right-handed patient diagnosed with pharmaco-resistant juvenile myoclonic epilepsy from the National Institute of Neurology and Neurosurgery at Mexico City, Mexico. Her past medical history includes atopy and frequent upper-airway infections, and an amygdalectomy at the age of 3 years old. She has no other relevant perinatal, psychiatric, or familiar past medical history.

**Figure 1:**
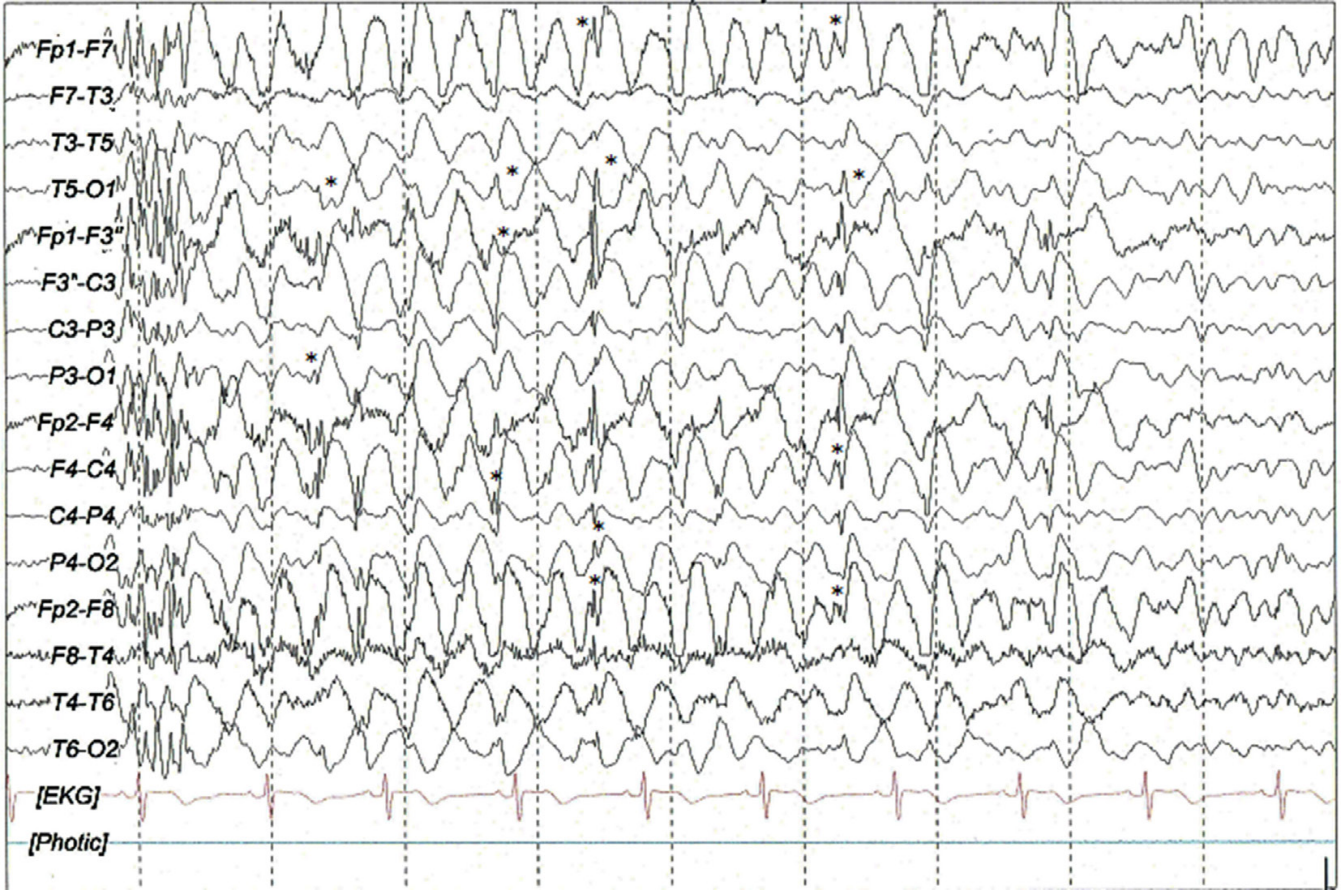
Interictal sleep (N1 stage) EEG scalp recording with generalized paroxysm of poly-spikes followed by fragmented 1–1.5 Hz spike and slow-wave complexes (*) mixed with slow waves at 3–4 Hz with higher amplitude in the anterior quadrants. Filters: 0.03–70 Hz, notch: 60 Hz, sensitivity: 7 μV/mm.

The patient presented her first seizure at age 12 years, describing this first episode as a generalized tonic–clonic seizure followed by myoclonic seizures. She was first seen in our institute 3 years after this initial event. During this 3-year interval, she was treated with valproate, topiramate, and lamotrigine, showing no improvement of her seizure frequency and developing pharmacoresistance to these antiepileptic drugs. Her seizure frequency was of 4 myoclonic seizures and 20 absence seizures per month, and only in rare occasion’s tonic–clonic seizures (once every 2–3 months). She denied any type of status epilepticus in the past. Her antiepileptic treatment at the moment of the intervention was levetiracetam (1 g/TID), carbamazepine (300 mg/BID), and pyracetam (800 mg/TID). The patient was informed about the tACS as a neuromodulation technique and was asked to participate in this intervention since she had pharmaco-resistant juvenile myoclonic epilepsy. After she signed the consent form to participate in this intervention, the patient was enrolled. The vital signs, neurological and psychiatric findings were normal. The psychological evaluation with the Barcelona Test (13) and Trail Making Test showed difficulties to store and recall visuospatial information, inability to complete cognitively complex tasks, fluctuations in sustaining attention, and difficulties in finding strategies, planning and performance, these features are related to the frontal lobe of the brain. Her brain 3-T magnetic resonance was normal, and the 1-h video-EEG showed interictal intermittent generalized paroxysms of spike-low wave at 3 Hz, polispike-slow waves at 3–4 Hz, and generalized slow rhythmic waves at 4 Hz with higher amplitude in the anterior quadrants (Figure 1). She also had several typical electro-clinical myoclonic and absence seizures during the video-recording. Basic medical laboratory studies (cell blood count, liver function test, blood electrolyte levels, glucose, and renal function test) were normal. Carbamazepine and pyracetam blood levels were in recommended therapeutic range.

### Intervention

The stimulation site was determined to be the most active epileptiform zone by EEG visual inspection. The EEG device was a Galileo (EBNeuro, Firenze, Italy) with golden cup electrode (Natus Medical Incorporated, Middleton, WI, USA) (Figure 1). The alternating current was applied over Fp1 and Fp2 (10–20 EEG international system) *via* a disposable stainless steel subdermal needle (Cardinal Health, Dublin, OH, USA) 12 mm in length and 0.4 mm in diameter using an Endeavor™ IOM Systems-^®^ (Natus Medical Incorporated, Middleton, WI, USA) as tACS. The stimulus consisted in a pulse train with a frequency of 3 Hz, 1 mA at 60 min during four consecutive days.

During the 4-day intervention period, the patient had a seizure-free lapse. At her 1-month follow-up, she reported an increase in seizure frequency characterized by two myoclonic seizures, four tonic–clonic seizures, and a myoclonic status epilepticus lasting 24 h, making it necessary to be treated at the emergency room with oral clonazepam (2 mg) and intravenous sodium valproate (1200 mg). We calculated an increase in seizure frequency as 75% since her basal frequency was of four myoclonic events per month during last 6 months. After the intervention, she presented a total of seven ictal events. We emphasize the fact that she had an increase in her generalized tonic–clonic seizures frequency and developed her first status epilepticus. No other cognitive deficits or neurological deficits were mentioned or found during medical evaluation.

At the 2-month follow-up, the patient stated that she had a 15-day seizure-free period. After this medical consultation, we lost contact with the patient.

## Discussion

We present the case of a female teenager with pharmaco-resistant juvenile myoclonic epilepsy who received tACS and developed a worsening of her epileptic syndrome including a myoclonic status epilepticus.

Epilepsy treatment options based on neurostimulation such as chronic intermittent vagal nerve stimulation (VNS) and responsive neurostimulation system based on deep brain and cortical stimulation (Neuropace, Mountain View, CA, USA) are approved by the Food and Drug Administration for the treatment of pharmaco-resistant focal epilepsy (14, 15). However, other non-invasive experimental neuromodulation techniques such as transcranial magnetic stimulation, deep brain stimulation (DBS), external trigeminal nerve stimulation, and tDCS have gained international medical attention in recent years (16). The underlying principle of these techniques relies on the idea that extrinsic stimulation can reduce hyperexcitability or interfere with the discharges of epileptogenic networks (11).

Vagal nerve stimulation, responsive neurostimulation system, DBS, and external trigeminal nerve stimulation devices work in different ways, but share a similarity in the fact that most of them uses frequencies above 10 Hz, thus causing an inhibitory effect (17). Nevertheless, none of these neuromodulation therapies have been approved in the treatment of pharmaco-resistant genetic generalized epilepsy, and there exists a limited experimental experience for the use of these techniques in the treatment of pharmaco-resistant genetic generalized epilepsy.

Transcranial alternating current stimulation is a non-invasive stimulation technique that modulates ongoing neural oscillation at specific frequencies using different current wave forms (18). The waveform of the stimulation changes cyclically over time, with either sinus pulses or square pulses that penetrate the skull through the electrodes placed over the surface of the scalp or are transmitted through the eye and optic nerve to the brain (19, 20). The effects of tACS at the neuronal level highly depend on the parameters used, i.e., current density, frequency range, electrode size, and the location of the stimulation electrode (21). Most of the applied stimulation frequencies are within the human EEG frequency range inducing local oscillatory activity in a stimulated brain area (22). In tACS, a frequency that matches the endogenous frequency could entrain network oscillations, and in our tACS protocol, we used 3 Hz similar to the frequency of the spike-slow waves of our patient (21). This effect is known as phase-locked waves (1). Previous alternating current stimulation (ACS) studies using several frequencies (1, 10, 15, 20, 30, and 45 Hz) during 5–10 min showed that only ACS delivered at 20 Hz over left motor cortex in healthy subjects induced increased excitability compared to other experimental protocols (23). However, combined ACS frequencies in delta (1–3 Hz), theta (5 Hz), alpha (10 Hz), and beta (20 Hz) ranges over the motor cortex in healthy subjects showed increased cortical excitability after theta–beta frequency stimulation, in comparison to alpha–alpha stimulation, the synergic mechanism is unknown (24). To the best of our knowledge, tACS has never previously been used in patients with genetic generalized epilepsy (21).

Ozen et al. tested tACS in an animal model using sinusoid patterns at slow frequency (0.8–1.7 Hz), recorded intracranial cortical neuronal activity, and found that tACS entrained 20 and 16% of neurons in the neocortical and hippocampal areas, respectively. These phase-locked neurons were intensity dependent. The intensities that effectively phase locked the spikes induced intracellular polarization values of 2–3 mA, suggesting that the stimulation intensity affected the number of spiking neurons recruited (25).

Alternating current stimulation-induced after effects are assumed to arise from synaptic-level processes (26), this long-lasting effect persists up to 1 h (8). In our patient, the adverse effects were reported at the first month of follow-up. Previous studies using tACS only reported minor side effects including light itching sensation under electrodes, tingling, a burning sensation, mild headache, nausea, and fatigue (27–30).

The adverse effects that are described in our patient by tACS could be explained due to the low stimulus frequency that was in an excitatory range, thus leading to an increase in the neural firing and synchrony which caused an increase in the seizures number and severity (8). Temporary modifications of the synapse once exposed to a rapidly alternating electrical field, alters the associated biochemical mechanisms, such as accumulation of calcium in the presynaptic nerve terminals leading to short-term synaptic plasticity effects thus causing an increase in neurotransmitter release (31).

Future clinical trials using tACS in generalized epilepsy potentially need to use frequencies higher than 10 Hz to modulate functional connectivity between the stimulated area and more distant but anatomically and functionally connected regions (32). Also, choosing the stimulation intensity is also a key consideration during tACS studies (33). Safety issues and long-term effects do need to be taken into consideration, as seen with our patient, at the 2-month follow-up she had a 15-day seizure-free period.

The limitations of our case report are the inherent to this type of studies, including the limited generalization of our findings to other type of genetic generalized epilepsies, lack of control of other unknown potential triggers of status epilepticus, and use of specific parameters of tACS.

## Author Contributions

DS-J, the main author, contributed in the redaction and final review of the manuscript once it was finished; he was also in charge of the study that enrolled the patient. CS, AH-R, EE-Z, GS-V, GR-A, and HZ-G were in charge of the manuscript redaction, application of tACS, and bibliographical search. CZ-J contributed in the understanding of the neuropsychological evaluation of the patient’s case.

## Conflict of Interest Statement

The authors declare that the research was conducted in the absence of any commercial or financial relationships that could be construed as a potential conflict of interest.
